# Generation and characterization of a tamoxifen-inducible lineage tracing tool *Cd2-P2A-CreERT2* knock-in mice

**DOI:** 10.3389/fimmu.2025.1482070

**Published:** 2025-03-10

**Authors:** Yang Guo, Mengyan Zhu, Zhilan Yu, Qing Li, Yanjuan Chen, Lei Ci, Ruilin Sun, Ruling Shen

**Affiliations:** ^1^ Model Organism R&D Department, Shanghai Laboratory Animal Research Center, Shanghai, China; ^2^ Shanghai Engineering Research Center for Model Organizations, Shanghai Model Organisms Center, Inc., Shanghai, China

**Keywords:** CD2, CreERT2/loxP system, tdTomato, tamoxifen-inducible, conditional gene manipulation

## Abstract

**Introduction:**

The new targeted gene editing technologies, such as the CRISPR/Cas system, enable researchers to insert or delete genes at targeted loci efficiently. The Cre-loxp recombination system is widely used to activate or inactivate genes with high spatial and temporal specificity.

**Methods:**

Using the CRISPR/Cas9 system, we inserted the *CreERT2* transgene expression cassette into the *Cd2* gene locus to generate conditional Cre-driver line *Cd2-CreERT2* knock-in mice, which drove the expression of CreERT2 by the endogenous *Cd2* promoter. By mating the *Cd2-CreERT2* strain with a *Rosa26-LSL-tdTomato* reporter mouse strain which contains a tdTomato expression fragment blocked with a loxP-flanked STOP cassette (LSL) driven by a CAG promoter, a *Cd2-CreERT2;Rosa26-LSL-tdTomato* reporter strain was obtained to evaluate the expression pattern of CD2 in different cell types.

**Results:**

After treatment with tamoxifen, the *Cd2-CreERT2* knock-in mice were induced to perform efficient recombination at the *loxP* site following CreERT2 activation and cause the expression of tdTomato fluorescence. The tdTomato and CD2 were expressed in the T cells of peripheral blood, spleen and mesenteric lymph nodes, whereas detected in a low proportion in the B cells. While about 20% of cells labeled with tamoxifen-induced tdTomato were CD2^+^ monocytes in peripheral blood, 10% of dendritic cells were tdTomato^+^/CD2^+^ cells. Tamoxifen-independent expression of tdTomato occurred in approximately 3% of CD2^+^ macrophages, but in negligible (~0.5%) in CD2^+^ granulocytes.

**Discussion:**

This work supplied a new transgenic mouse as a valuable tool for lineage tracing in CD2-expressing cells, for conditional mutant studies of immune modulatory effects in a time-dependent manner, and analysis of the potential therapeutic effect of CD2-targeting biologics.

## Introduction

Cre recombinase produced by bacteriophage P1 enables efficient editing of DNA with *loxP* sequence, both *in vivo* and *in vitro (* 1). Transgenic mice with specific sequence flanked with *loxP* (termed “floxed”) are usually crossed with mouse lines expressing Cre recombinase to perform a lineage-specific or inducible mannered gene expression (or inhibition) (2, 3). To realize the specific spatiotemporal regulation of gene recombination, Cre recombinase was fused with the ligand binding domain of the human estrogen receptor (ER) containing three mutations (C400V/M453A/L544A), to form the fusion protein as CreERT2 recombinase (4). Following activation by the synthetic estrogen-like agonist tamoxifen or its active metabolites 4-OH-tamoxifen, CreERT2 dissociates from the anchor protein HSP90 via a conformational change, enters the nucleus, recognizes *loxP* sites, and recombines the floxed region (4). Due to the characteristics of various tissue/cell type-specific Cre or tamoxifen-inducible CreERT2-driver lines and floxed alleles (5–9), Cre is introduced into the genome by regular transgenesis or inserted into defined loci to give the expected expression profile (10, 11).

CD2 is a costimulatory protein expressed mainly in T/NK cells (12), thymocytes (13), dendritic cells (14) and also B cells (12, 14–16). For several decades, CD2 has been thought to be involved in T cell activation (17, 18). The multifunctionality of CD2 is considered to involve a) contribution to mediate cell adhesion, b) accumulation in the immunological synapse (IS), and c) recruiting the intracellular kinases to the IS (19). The upregulated expression of CD2 can be detected in activated T cells and memory T cells (20, 21), as well as in NK cells, considered contributing to triggering a series of intracellular signaling between NK cells and their target cells (22, 23). High expression of CD2 is also a marker for mouse and rat spermatogonial stem cells (SSCs) (24). It is suggested that CD2-targeted therapies may be useful for the treatments of these cell types-specific diseases and autoimmunity in humans.

Several human *CD2* promoter driver Cre transgenic mouse lines (*hCD2-iCre*) have been reported and characterized. These can be used to assess the function of the gene or cells through efficient genetic manipulation (25–27). However, *hCD2*-knockin and Cre-mediated deletion of *hCD2-iCre* lines exhibit flaws with regard to the recombination efficiency or disruption of mouse endogenous CD2 expression. To assess the activity pattern of this important *Cd2-Cre* line with cell-specific phenotype, we generated a new *Cd2^P2A-CreERT2^* knock-in line (*Cd2-CreERT2*), in which the *P2A-CreERT2* sequence was inserted upstream of the stop codon at the *Cd2* locus. Through crossed with a *Rosa26-LSL-tdTomato* line which contains a tdTomato expression fragment blocked with a *loxP*-flanked STOP cassette (LSL) driven by a CAG promoter and induced with tamoxifen, the *Cd2-CreERT2* driver line enabled efficient and specifical removal of the floxed stop signal in expected cell lines without causing Cre-mediated recombination in no-Tamoxifen or no Cre driver lines. The crossed line (named *Cd2-CreERT2;Rosa26-LSL-tdTomato*) showed no evidence of disruption in endogenous *Cd2* expression, which allowed it to be used to evaluate the expression of CD2 in different cell types of different tissues directly. Flow cytometric assay and immunofluorescence staining were further employed to characterize the tdTomato/CD2 expressed in the cells of different lineages. Altogether, we aimed to explore that how the new *Cd2-CreERT2* line could be useful for performing specific genetic manipulations in mice, and for further studies of gene function in different cell types.

## Materials and methods

### Animals

All animals used come from the Shanghai Laboratory Animal Research Center. Animal procedures were performed in accordance with the regulations of the Shanghai Laboratory Animal Research Center and the Institutional Animal Care and Use Committee (authorization number: 202301902). All mice were maintained in a specific pathogen-free facility under a 12-12h light/dark cycle with *ad libitum* access to food and water.

### Generation of C57BL/6-*Cd2^P2A-CreERT2^* knock-in mice

C57BL/6J-*Cd2^P2A-CreERT2^* knock-in mice (abbreviated as *Cd2-CreERT2*) developed by CRISPR/Cas9-mediated gene editing in zygotes, were generated by Shanghai Model Organisms Center, Inc. (SMOC Shanghai, China).

To generate the *Cd2-CreERT2* knock-in mice, a donor DNA template was constructed, containing the sequences of the 5’ homology arm of the insertion site (4.3kb), a self-cleaving peptide porcine teschovirus-1 2A (*P2A*), the *CreERT2* sequences, a woodchuck hepatitis post-transcriptional regulatory element (*WPRE*), an exogenous polyadenylation (polyA) signal sequence, and the 3’ homology arm of the insertion site (2.5 kb). The sequences were PCR-amplified from existing plasmids and the C57BL/6J mouse genome using the Phusion high-fidelity DNA polymerase (New England Biolabs, Ipswich, MA, USA). The pBR322 plasmid (Stratagene, La Jolla, CA, USA), linearized with SacII digestion, was used as the backbone of the donor vector. The In-Fusion HD Cloning Kit (Clontech, Mountain View, CA, USA) was used to fuse the sequences and generate the vector. The insertion site of the transgene cassette was designed at the direct upstream of the stop codon of *Cd2*. The CRISPR/Cas9 system was employed to perform a cleavage around the insertion site and lay the condition for the subsequent homology dependent repair. The guide RNA (gRNA) of the Cas9 system, targeting the CTGCCGCCCCCTAATTAAGA sequence in the *Cd2* exon5, was synthesized using HiScribe™T7 High Yield RNA Synthesis Kit (E2040S, New England Biolabs France). Cas9 mRNA was generated *in vitro* using mMESSAGE ^®^ T7 Ultra Kit (AM1345, Thermofisher). The event of genomic DNA being introduced with the transgene is schematically illustrated in **Supplementary Figure 1A**.

The zygotes from the C57BL/6J strain were injected with the mixture of the gRNA, *Cas9* mRNA and purified donor vector DNA. Knock-in founders (the F0 generation) and the offspring F1 generation were screened by PCR genotyping analysis with their genomic DNA extracted from the tails. The F0/F1 PCR genotyping strategy is shown in **Supplementary Figure 1B**. The primers used for the F0/F1 genotyping are shown in **Table 1**.

**Table 1:** PCR primers for amplifying and sequencing CRISPR/Cas9-induced modifications.

Primer	Sequences (5’-3’)
P1	GGAAGACTAGAGGGACAGAAGTAACAAC
P2	TGACCAGAGTCATCCTTAGCG
P3	GCACTGTTTTCCTGTTACTCTTCCCTA
P4	GAACCTCACCAATCCCATCCTTT

The PCR genotyping for the F2 and later generations was refined to amplify shorter fragments, as the strategy shown in **Supplementary Figure 2A**, the primers and PCR condition shown in **Table 2**.

**Table 2:** PCR information.

Primer	Sequences (5’-3’)	Cycling Reaction	Product size
P5	CCATCACCTCCAGACACCT	94 °C 5min 94 °C 30s, 58 °C 30s,72 °C 30s (35 cycle) 72°C,5min	WT: 366bp KI: 410bp
P6	GCCAGACCAAGTGACTGTG		
P7	GGCAAACGGACAGAAGCAT		

min, minutes; s, seconds; bp, base pairs; WT, wild-type fragment; KI, knock-in fragment

### Production of *Cd2-CreERT2;Rosa26-LSL-tdTomato* reporter mice

The *Cd2-CreERT2* mice were crossed with a *loxP*-flanked STOP cassette (LSL) *Rosa26-LSL-tdTomato* reporter line (AI9, Jackson Labs) (28) to yield a *Cd2*-driven tdTomato reporter mice (*Cd2-CreERT2;Rosa26-LSL-tdTomato*).The genotype of *Cd2-CreERT2;Rosa26-LSL-tdTomato* mice was identified using the same strategy as genotyping of *Cd2-CreERT2* F2 generation for the *Cd2* locus, and the protocol given by Jackson Labs for the *Rosa26* locus. Mice heterozygous at both loci and aged 6 ~ 8 weeks were used for further experiments.

### Tamoxifen treatment

The novel *Cd2-CreERT2;Rosa26-LSL-tdTomato* mice were treated with tamoxifen (SigmaAldrich, St. Louis, MO, USA) to induce CreERT2-mediated recombination. The tamoxifen was dissolved in corn oil (20 mg/ml) and shaken overnight at 37°C. Each adult mouse was injected 5 times intraperitoneally (*i.p*) with 120mg/kg body weight of tamoxifen/corn oil once every two days (on day 0, 2, 4, 6, 8) (29). These mice were anaesthetized on the 7th day following the final injection and examined for the efficiency of CreERT2-mediated recombination of the *loxP-*flanked STOP cassette at the tdTomato locus.

### Flow cytometric analysis

The thymus, mesenteric lymph node, spleen and bone marrow were harvested and processed into single-cell suspensions. A total of 1 × 10^6^ cells were incubated with fluorescent antibodies. The cells were resuspended in 100 μl PBS and analyzed on a CytoFlex flow cytometer (Beckman, Indianapolis, United States). All antibodies were used at appropriate saturating concentrations. The following antibodies were purchased from BioLegend: anti-CD45-BV605 (30-F11), anti-CD3-FITC (17A2), anti-CD8-PC5.5 (53-6.7), anti-CD4-BV421 (GK1.5), anti-NK1.1-PC5.5 (PK136), anti-CD19-APC (6D5), anti-IgD-FITC (11-26c.2), anti-Gr1-BV510 (RB6-8C5), anti-CD11b-PE-CY7 (M1/70), anti-CD11c-BV421 (N418), and anti-CD2-APC (RM2-5). tdTomato expression was determined by the PE channel. Gates were set up according to the unstained controls.

### Tissue trangenic fluorenscence imaging

The brain, lung, heart, thymus, spleen, kidney, pancreas, liver, stomach, intestine, mesenteric lymph node, and gonad were harvested from the mice. The tdTomato fluorenscence pictures of the tissues were captured with a live animal imaging system (AniView100, Guangzhou Biolight Biotechnology Co.). The thymus, spleen and mesenteric lymph nodes were further imaged with a stereo microscope equipped with epi-fluorescence attachment (SMZ1000, Nikon). With either equipment, the fluorescence images were captured under identical imaging conditions.

### Immunofluorescence staining and imaging

Mouse thymus, spleen and mesenteric lymph nodes were harvested and washed with ice-cold PBS. The samples were fixed with prechilled 4% paraformaldehyde, equilibrated in sucrose-PBS at concentrations from 15% to 30%, embedded in Tissue Freezing Medium OCT (CM1950, Leica), frozen and then cryosectioned (5 μm). The cryostat sections were washed with PBS and then permeabilized and blocked with blocking solution (5% donkey serum, 017-000-121, Solarbio; 0.1% Triton X-100, A110694-0100, Sangon Biotech; in PBS) for 1 hour at room temperature. The sections were incubated with primary antibody (donkey anti-rat CD2 antibody, 1:200 dilution, sc-53046, Santa Cruz) overnight at 4°C, then with fluorochrome-conjugated secondary antibodies (Alexa Fluor 488, A-21206, Thermofisher) for 1 hour at room temperature. The sections were mounted with VECTASHIELD^®^ Antifade Mounting Medium with DAPI (H-1200, Vector Labs). The tdTomato fluorescence was visualized without immunostaining. A confocal microscope (LSM 710, Zeiss) was used to capture the flourescence images.

### Quantitative RT-PCR analysis

Quantitative RT-PCR analysis was performed to confirm the effect of knock-in transgene cassette on endogenous *Cd2* expression using TransStart^®^ Top Green qPCR SuperMix (AQ131, Transgen, Beijing, China). The primer sequences used for each target gene: *Cd2*, 5’- TGGTGTATGGCACAAATGGG -3’ and 5’- GCTTTGAGACCCTCTCCAGAAT -3’; *β-actin*, 5’- GGCTGTATTCCCCTCCATCG -3’ and 5’- CCAGTTGGTAACAATGCCATGT-3’.

### Western blot analysis

Western blot was performed using established procedures with indicated primary antibodies. Briefly, the protein in spleen homogenate was extracted with RIPA lysis buffer (Takara, Dalian, China). The protein was separated on 4% ~ 20% polyacrylamide gel and electro-transferred to nitrocellulose (NC) membrane. 5% BSA was used to block NC membranes for 1 h. The membrane was incubated with primary antibodies to CD2 (ab219411, Abcam, Cambridge, UK) and GAPDH overnight at 4°C, then with Donkey anti-Rabbit secondary antibodies (LI-COR, Lincoln, Nebraska, United States) for one hour at room temperature. Then the membrane was visualized using Odyssey Imaging System (Odyssey, Shenzhen, China).

### Southern blot analysis

The Southern blot analysis was performed to confirm the proper insertion of the *CreERT2* transgene cassette. The genomic DNA of the mice was digested with the restriction enzyme BglII or AflII, resulting in the *CreERT2* knock-in allele to be probed digested into an 8.8 kb fragment or a 6.6 kb fragment. A 323-bp probe named Probe-Cre was used to detect the *CreERT2* sequence. The sequence of Probe-Cre: ACGTATAGCCGAAATTGCCAGGATCAGGGTTAAAGATATCTCACGTACTGACGGTGGGAGAATGTTAATCCATATTGGCAGAACGAAAACGCTGGTTAGCACCGCAGGTGTAGAGAAGGCACTTAGCCTGGGGGTAACTAAACTGGTCGAGCGATGGATTTCCGTCTCTGGTGTAGCTGATGATCCGAATAACTACCTGTTTTGCCGGGTCAGAAAAAATGGTGTTGCCGCGCCATCTGCCACCAGCCAGCTATCAACTCGCGCCCTGGAAGGGATTTTTGAAGCAACTCATCGATTGATTTACGGCGCTAAGGATGACTCTG. The localization of the restriction enzyme sites and the probe on the genome map is shown in **Supplementary Figure 3A**.

### Statistical analysis

Comparisons of significant differences were done by Students’ t-test when compared with corresponding control or by ANOVA followed by Sidak’s or Dunnett’s multiple comparisons test as indicated using Prism 8 software (*p < 0.05; **p < 0.01; ***p < 0.001; ****p < 0.0001).

## Results

### Generation and characterization of *Cd2-CreERT2* knock-in mice

A CRISPR/Cas9 strategy was designed to insert *CreERT2*, preceded by *P2A*, into the stop codon of the murine *Cd2* locus, thereby enabling the transgene to be driven by the endogenous *Cd2* expression. As the result of zygote microinjection, 6 F0 mice were generated, then both of them crossed with the C57BL/6J mice to obtain 7 F1 mice, of which 5 mice obtained from 4^#^ F0 male mouse. The heterozygous *Cd2-CreERT2* mice showed no abnormalities in embryo development or their growth and reproductive ability. In the PCR genotyping of the F1 generation, a 7.3 kb fragment was amplified from the wild-type allele, so as a 2.9 kb fragment from the knock-in allele with the P3/P4 primers; a 5.8 kb fragment was amplified from the knock-in allele with the P1/P2 primers (**Supplementary Figure 1C**). The primers P1 and P4 were positioned outside the homologous arms. The primer P3 sequence was a segment of exon 4, and had been pre-inserted on donor vector at the end of WPRE-polyA to facilitate subsequent screening. In the PCR genotyping of the F2 generation, a 366 bp fragment was amplified from the wild-type allele with the P5/P6 primers; a 410 bp fragment was amplified from the knock-in allele with the P5/P7 primers (**Supplementary Figure 2B**). The heterozygous *Cd2-CreERT2* knock-in mice were bred for at least four generations prior to starting any experiments.

To confirm the insertion count of the *Cd2-CreERT2* strain, the wild-type or heterozygous knock-in mice screened by PCR were further analyzed by southern blot. No probed bands were detected in the wild-type mice (No. 273, 242). Unique bands of expected length were detected in the knock-in mice (No. 276, 253, 243), indicating that a proper single copy of the transgene cassette was inserted at the targeted locus (**Supplementary Figure 3B**).

The *Cd2* mRNA and protein expressions in the spleen of heterozygous *Cd2-CreERT2* mice and wild-type C57BL/6 mice were evaluated by quantitative RT-PCR and western bloting. The endogenous *Cd2* expression level of the heterozygote was not affected by transgene insertion (**Supplementary Figure 4**). The subcellular localization of CD2 in the mesenteric lymph nodes of the homozygous *Cd2-CreERT2* mice was confirmed by immunofluorescence section images. No abnormality in the subcellular localization of CD2 was observed in the lymphocytes (**Supplementary Figure 5**).

### *Cd2-CreERT2* knock-in leads to efficient recombination

**Figure 1:**
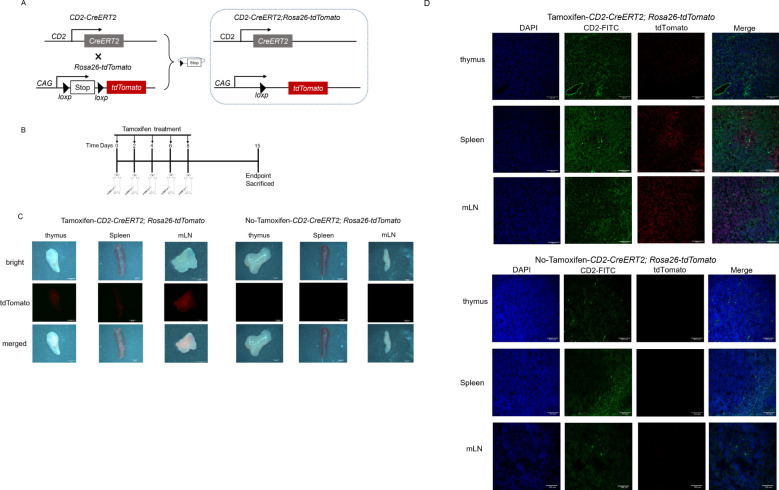
Cre recombinase-expression and fluorescence signal in a *Cd2-CreERT2*-inducible tdTomato mouse model. **(A)** Schematic diagram depicting the *Cd2-CreERT2* mouse crossbred with a tdTomato reporter mouse line. **(B)** Schematic diagram depicting the Tamoxifen-induced Cre-recombinase. **(C)** Representative images of thymus, spleen and mesenteric lymph node (mLN) from *Cd2-CreERT2;Rosa26-LSL-tdTomato* mice visualized directly for tdTomato fluorescence. **(D)** Representative immunofluorescence images of tissue sections for tdTomato expression in CD2^+^ cells (green).

To determine the recombination efficiency of *Cd2-CreERT2*, we used the STOP-floxed *Rosa26-LSL-tdTomato* reporter line (*Rosa26-LSL-tdTomato* mice) to test the activation of the reporter. Heterozygous *Cd2-CreERT2* mice were mated with heterozygous *Rosa26-LSL-tdTomato* mice to obtain *Cd2-CreERT2;Rosa26-LSL-tdTomato* mice (**Figure 1A**). Adult *Cd2-CreERT2;Rosa26-LSL-tdTomato* mice were administered tamoxifen on days 0, 2, 4, 6 and 8 (**Figure 1B**). One week after the last treatment, Cre activity in major organs was examined through tdTomato signal intensity, either by direct visualization of organs using stereoscopic fluorescence microscopy or by immunofluorescence stained cryosections. Direct visualization revealed strong fluorescent signals in the mesenteric lymph nodes of *Cd2-CreERT2;Rosa26-LSL-tdTomato* females and males (**Figure 1C**). No tdTomato signal other than the nonspecific fluoroscence in stomach and intestine was detected in the absence of tamoxifen treatment in male *Cd2-CreERT2;Rosa26-LSL-tdTomato* mice (**Supplementary Figure 6**). While the female *Cd2-CreERT2;Rosa26-LSL-tdTomato* mice showed minor tdTomato signal in brain, thymus, pancreas, liver and uterus without tamoxifen treatment, the intensity was proximate to the lowest value on the scale bar and as closed to non-specific fluorescence of comparable intensity detected in the stomach and intestine of the wild-type mice, which hinted not be an exact tdTomato signal in transgenic mice. CD2-labeled cells in the spleen were located in the red and white pulp near the marginal zone (**Figure 1D**). However, only red pulp expressed a high level of tdTomato (red). tdTomato/CD2 double-positive cells were observed in mesenteric lymph nodes in the Cre lines, while other tdTomato/CD2 subsets with weaker signals were observed in the thymus of Cre lines (**Figure 1D**).

**Figure 2:**
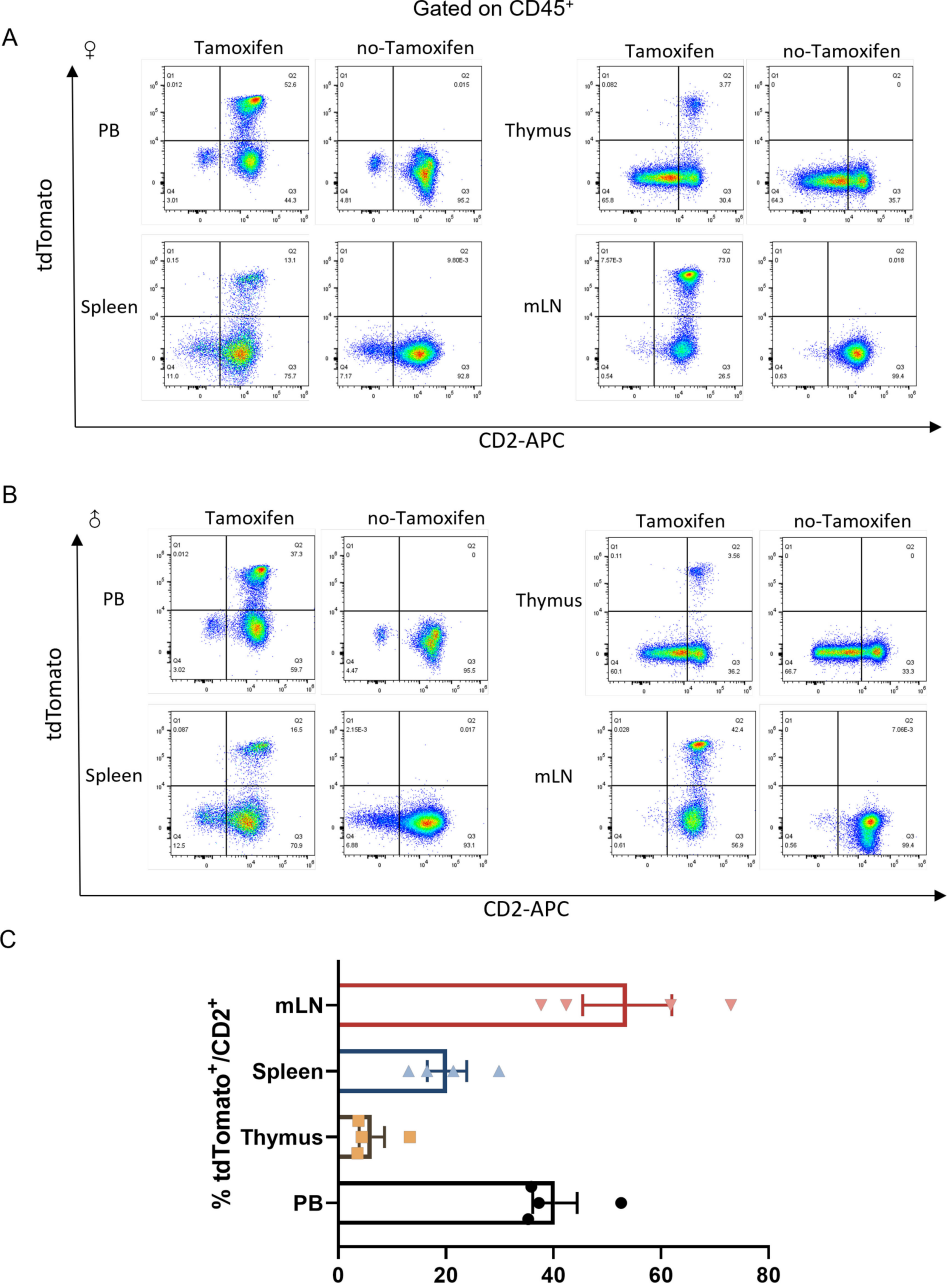
tdTomato^+^/CD2^+^ cells analysis from flow cytometry measurements. Representative flow cytometry plots of tdTomato^+^/CD2^+^ cells from CD45^+^ subpopulations of peripheral blood (PB), thymus, spleen (SP) and mesenteric lymph node (mLN) in *Cd2-CreERT2;Rosa26-LSL-tdTomato* female **(A)** or male **(B)** mice with or without tamoxifen treatment. **(C)** Quantification of tdTomato^+^/CD2^+^ % cells, n=4.

The recombined cells stained with CD2 antibody were analyzed by flow cytometry. Stable tdTomato expression with higher CD2 expression was found in peripheral blood, spleen and mesenteric lymph node, but lower expression was observed in the thymus (**Figure 2**). This was similar to a previous report on a validated *hCD2-iCre^+/-^Rosa26-stop-EYFP^+/-^* reporter line (27). We could not find any tdTomato-labeled cells in any tissues from *Cd2-CreERT2;Rosa26-LSL-tdTomato* mice without tamoxifen treatment, suggesting no Cre recombination leakage in the reporter line.

We therefore concluded that a tamoxifen-inducible CreERT2 recombinase line was obtained and could be used to reveal endogenous *Cd2* promoter activities.

### Cre activity is specifically induced in T cells

**Figure 3:**
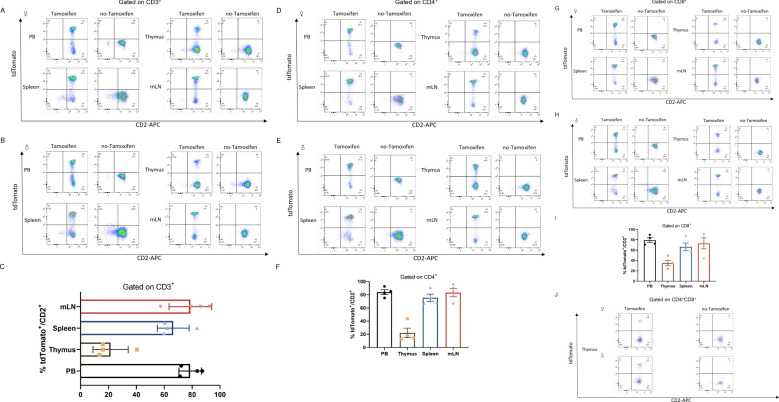
CD2-Cre activity in mature leukocytes (T cells). Co-expression of CD2 and tdTomato in T cells from peripheral blood (PB), thymus, spleen (SP) and mesenteric lymph node (mLN) gated on CD3^+^ cells from *Cd2-CreERT2;Rosa26-LSL-tdTomato* female **(A)** or male **(B)** mice with or without tamoxifen treatment **(A, B)** and graph bar **(C)**. **(D–F)** gated on CD4^+^ cells from *Cd2-CreERT2; Rosa26-LSL-tdTomato* female **(A)** or male **(B)** mice with or without tamoxifen treatment **(D, E)** and graph bar **(F)**, **(G–I)** Gated on CD8^+^ cells from *Cd2-CreERT2;Rosa26-LSL-tdTomato* female **(A)** or male **(B)** mice, with or without tamoxifen treatment **(G, H)** and graph bar **(I)**. **(J)** Co-expression of CD2 and tdTomato in CD4^+^CD8^+^ double-positive (DP) T cells from the thymus of *Cd2-CreERT2;Rosa26-LSL-tdTomato* female or male mice with or without tamoxifen treatment. n=4.

We next quantified the cell types that had undergone Cre-mediated recombination from the total cell population within the positive organs described above. To further investigate how Cre recombination occurred in different cell types, we also examined the percentage of total CD3^+^ T cells as developing T cells (**Figure 3**). As expected, a high percentage of tdTomato^+^ to total lymphocytes was observed in the peripheral blood (78.63 ± 4.041%), spleen (66.48 ± 5.701%) and mesenteric lymph node (78.73 ± 7.648%) respectively, but was less proportion in the thymocytes which are abundant CD2 population (21.53 ± 6.318%).

We examined the CD2-Cre at different T subtype cells. T cells originate from hematopoietic stem cells in bone marrow. T cell progenitors migrate to the thymus, where they undergo development and maturation. In the thymus, they sequentially progress through the double-negative (DN) stage and the CD4^+^CD8^+^ double-positive (DP) stage before differentiating into single-positive (SP) cells. These mature single-positive T cells subsequently migrate to peripheral organs, where they can become further activated and perform their functions upon antigen stimulation. Analysis of tdTomato expression in SP cells from *Cd2-CreERT2;Rosa26-LSL-tdTomato* mice showed a similar expression profile. A detectable amount of tdTomato did observe in the peripheral (83.98 ± 3.606%), spleen (75.48 ± 5.497%) and mesenteric lymph node (83.23 ± 6.220%) respectively. Consistent with previous reports (30, 31), we found tdTomato/CD2 expression (<20%) in DP stages of T cells in the thymus of heterozygous treated with five injects of tamoxifen (**Figure 3J**). Potential tdTomato/CD2 expression, or leakage to T cells was also evaluated. No fluorescent signal was observed in *Cd2-CreERT2;Rosa26-LSL-tdTomato* mice with no-tamoxifen, indicating the line is not leaky (**Figure 3**). This finding suggests that Cre activity is localized to these cell subsets, or had been localized at an earlier developmental time point.

### Cre activity is specifically induced in B cells

**Figure 4:**
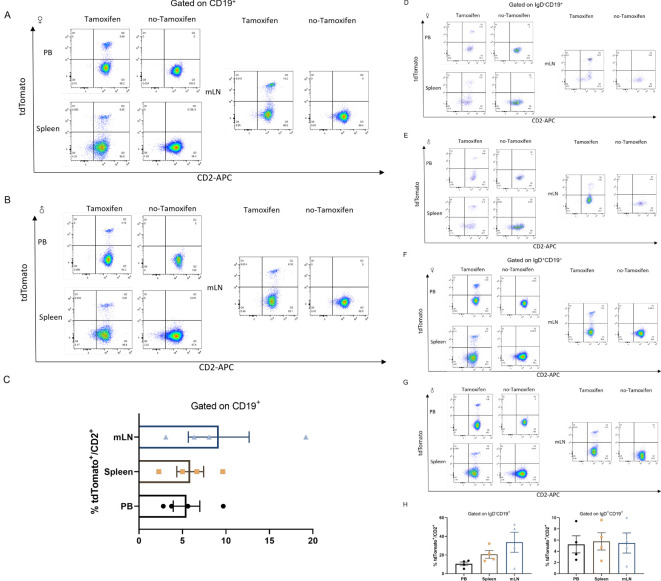
tdTomato signal in B leukocyte populations. Numbers depict the percent of CD2^+^tdTomato^+^ cells in *Cd2-CreERT2; Rosa26-LSL-tdTomato* female **(A)** and male mice **(B)** in peripheral blood (PB), Spleen (SP) and mesenteric lymph node (mLN) gated on CD19^+^ and the graph bar **(C)**. **(D–H)** Gated on CD19^+^IgD^-^ **(D, E)**, CD19^+^IgD^+^ **(F, G)** and graph bar **(H)** n=4.

Murine B cells also exhibit expression of CD2 (32). B cells develop through a series of stages in the bone marrow (BM) (33). All peripheral B cells and BM-derived IgD^-^CD19^+^ immature B cells displayed *hCD2-iCre* promoted EYFP expression, with high *Cd2* mRNA expression observed in post-fraction B cell developmental phases and mature B cells, while low *Cd2* mRNA expressed in hematopoietic stem cells (HSC) and hematopoietic progenitor cell (HPC) populations (27). IgD^+^CD19^+^ cells are mature recirculating B cells, whereas IgD^-^CD19^+^ cells represent a stage of B cell development prior to migration into the periphery. To investigate the stage of B cell development at which Cre expression initiates in this lineage, cells were isolated and stained with anti-IgD and anti-CD19 antibodies. The total number of tdTomato^+^ cells in B cells and their developmental stage were quantified by flow cytometry analysis. Interestingly, analysis of the *Cd2-CreERT2;Rosa26-LSL-tdTomato* line revealed a slightly different pattern of B cell development (**Figure 4**). A weak signal was detected in total CD19^+^ B cells from peripheral blood (5.463 ± 1.528%), spleen (5.895 ± 1.540%) and mesenteric lymph node (9.173 ± 3.498%) (**Figures 4A, B**). In contrast, stronger signals were found in the earliest B cell progenitors, IgD^-^CD19^+^ single positive, in mesenteric lymph node-derived B cells (33.68 ± 10.78%), compared to peripheral blood (10.48 ± 1.976%) and spleen (20.68 ± 4.152%) (**Figures 4D, E**). This result indicates that even the very few immature B cells had undergone Cre-mediated recombination. Other tdTomato/CD2 were also labeled in IgD^+^CD19^+^ double positive (DP) peripheral-derived B cells (**Figures 4F, G**). No substantial tdTomato expression was seen in B cells or development stages from *Cd2-CreERT2;Rosa26-LSL-tdTomato* transgenic mice that had not been induced with tamoxifen (**Figure 4**).

### Cre expression in NK cells

**Figure 5:**
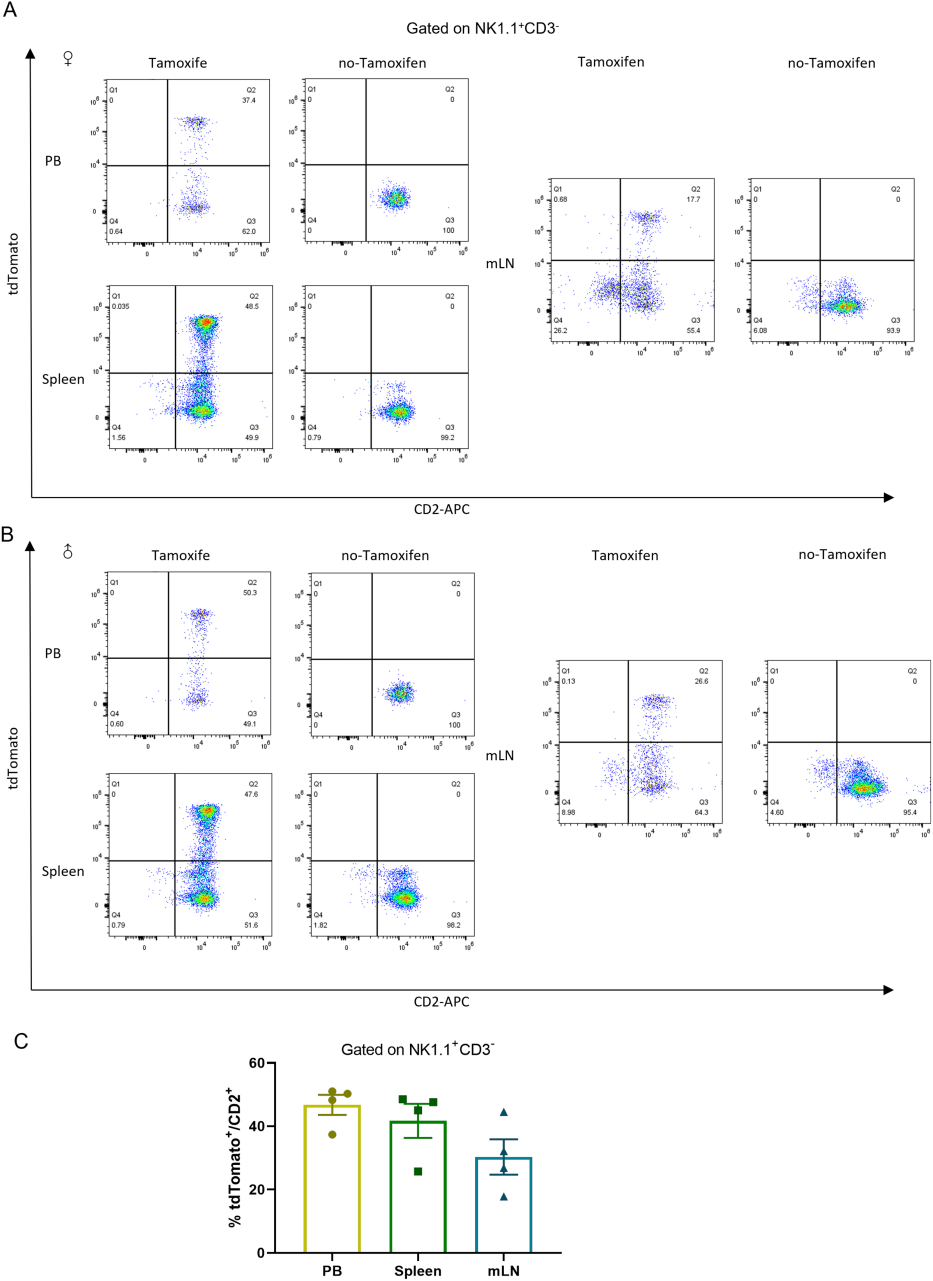
tdTomato signal in NK leukocyte populations. Numbers depict the percent of CD2^+^tdTomato^+^ cells in *Cd2-CreERT2; Rosa26-LSL-tdTomato* female **(A)** and male mice **(B)** in peripheral blood (PB), Spleen (SP) and mesenteric lymph node (mLN) gated on NK1.1^+^ CD3^-^ and the graph bar **(C)** n=4.

The CD2^+^tdTomato^+^ double positive cells were also identified in NK cells, which were characterized in NK1.1^+^CD3^-^ (33–35) double labeled NK cells populations in Tamoxifen-induced *Cd2-CreERT2;Rosa26-LSL-tdTomato* mice (**Figure 5**). About half of CD2-positive cells were labeled with tdTomato in NK1.1^+^CD3^-^ cells from the peripheral blood and spleen, while ~20% in NK1.1^+^CD3^-^ cells from the mesenteric lymph node (mLN). No substantial tdTomato expression was detected in NK cells in no-Tamoxifen treated *Cd2-CreERT2;Rosa26-LSL-tdTomato* transgenic mice.

### Cre expression in other lineages

**Figure 6:**
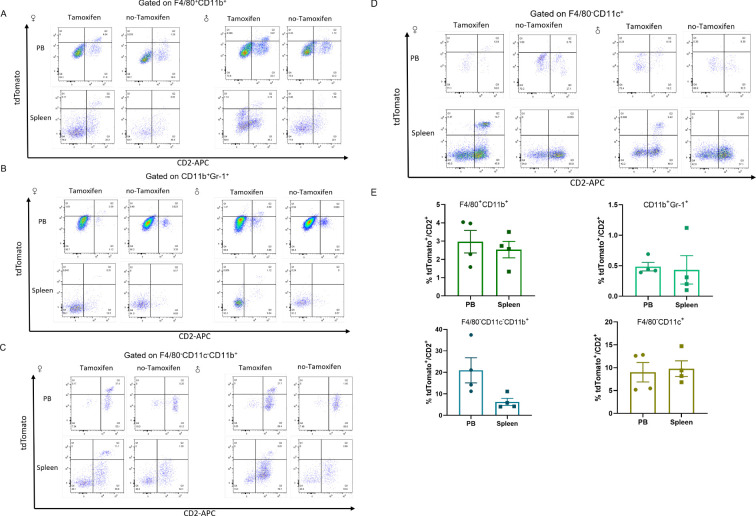
tdTomato expression in the indicated myeloid populations from *Cd2-CreERT2;Rosa26-LSL-tdTomato* mice. Flow cytometric analysis of tdTomato expression in macrophages **(A)**, granulocytes **(B)** monocytes **(C)** and dendritic cells **(D)** lineages. **(E)** The proportion of tdTomato^+^/CD2^+^ was charted as mean ± SEM, n = 4.

CD2 has been previously confirmed to drive transgene expression in all hematopoietic cells (36). To further confirm, the cells were probed with anti-CD11b and anti-F4/80 to mark macrophages (37), and with anti-Gr-1, anti-CD11b and anti-CD11c to identify granulocytes and monocytes (**Figure 6**). Approximately 3% and 2.5% tdTomato^+^/CD2^+^ cells were identified in macrophages (F4/80^+^CD11b^+^) from peripheral blood and spleen, respectively, in Tamoxifen-Cre lines (**Figure 6A**). Only 0.5% of CD2-positive cells were labeled with tdTomato in granulocytes (CD11b^+^Gr-1^+^) (**Figure 6B**). tdTomato^+^ cells were observed in F4/80^-^CD11c^-^CD11b^+^ cells (monocyte enriched) from the peripheral blood and spleen. Quantitative analysis revealed that 20.98 ± 5.854% of CD2^+^ cells in peripheral blood were tdTomato-positive, while 6.283 ± 1.655% were positive in the spleen (**Figure 6C**). These results indicate that CreERT2-mediated recombination occurred in monocytes. Approximately 10% of dendritic cells (F4/80^-^CD11c^+^) from peripheral blood and spleen, were tdTomato/CD2 positive cells (**Figure 6D**).

Collectively, the above results confirmed that *Cd2-CreERT2* activated by tamoxifen inducement performed Cre recombination with a myeloid linage manner in *Cd2-CreERT2;Rosa26-LSL-tdTomato* mice.

## Discussion

In the current study, the *Cd2* gene was targeted with knock-in *CreERT2* using the CRISPR/Cas9 system. This enabled the construction of *Cd2-CreERT2;Rosa26-LSL-tdTomato* mice for gene manipulation of lymphoid and myeloid cells.

CRISPR/Cas9 technology to generate transgenic mice to knock in a *P2A-CreERT2* sequence in exon5 of the endogenous *Cd2* gene. Utilized the endogenous *Cd2* promoter to trigger CreERT2 expression on the surface of T cells, NK cells, thymocytes and dendritic cells (38, 39). Because the P2A sequence-connected *CreERT2* gene was knocked in just before the *Cd2* stop codon in *Cd2-CreERT2*, the *Cd2* gene itself was not disrupted. It has been confirmed by quantitative RT-PCR and western blot analysis that the endogenous *Cd2* expression was not affected by the transgene insertion. The immunofluorescence section showed no abnormality in the subcellular localization of CD2 protein. Furthermore, CreERT2 expression leads to efficient recombination with tamoxifen administration when crossbred with *Rosa26-LSL-tdTomato* reporter mouse line containing STOP-floxed alleles. The dosage of tamoxifen used in this study did not show any significant toxicity (40).

Multiple confirmations showed correct activation of *Cd2-CreERT2*, no effect on *Cd2* expression, and normal basic morphology of specific cells in the transgenes. The genome editing techniques would cause the random insertion effect concerned. In this regard, we performed Southern blot analysis to confirm the precise insertion of the target sequence into the intended site (**Supplementary Figure 3**). Of note, the *Cd2-CreERT2;Rosa26-LSL-tdTomato* mice have no phenotypical changes due to heterozygosity. Immunofluorescence showed that tdTomato was expressed in the thymus, spleen and mesenteric lymph node (**Figure 1**).

The tdTomato was expressed almost exclusively in CD45^+^ cells (**Figure 2**). We further examined the expression of tdTomato and the activity of CreERT2 in different cell types from various tissues following the administration of tamoxifen. FACS indicated that tdTomato was observed in virtually all CD3^+^ T cells from lymphoid tissues such as the peripheral blood, thymus, spleen and mesenteric lymph nodes (**Figure 3**). The small proportion of positive cells not detected possibly reflected Cre-driven recombination efficiency, rather than cells that did not express CD2. Only a few CD4^+^CD8^+^ double positive-derived T cells from the thymus expressed tdTomato. In addition, we showed that CD19^+^ B cells also expressed tdTomato and displayed CreERT2 activity. About 10% tdTomato^+^ cells over total cells in mLN were found in *Cd2-CreERT2;Rosa26-LSL-tdTomato* mice. This contrasts with previous studies that found high hCD2-iCre activity (>83% EYFP^+^) in CD4^+^CD8^+^ thymocytes, and >95% EYFP^+^ in all B cell developmental stages. This could be because in the present study, *Cd2-CreERT2* utilized an endogenous promoter rather than an exogenous promoter. Previous studies have identified an impact of haploinsufficiency on phenotype in global Gcg null mutant mice (41), indicating insufficient transgene activation in specific cell types.

The transgenic *hCD2* line was reported to exist in immature B cells (30). Similarly, CD2 is also expressed in murine B cells (36). Supporting this view, CD19^+^IgD^-^ single positive immature B cells expressed higher levels of the reporter gene, especially in mesenteric lymph nodes. The *Cd2-CreERT2;Rosa26-LSL-tdTomato* in this study labels a few tdTomato in CD19^+^IgD^+^ mature B cells (<10%) (**Figure 4**). In addition to higher tdTomato levels in immature CD2^+^ B cells, which could be due to CD2 being active during the early stages of B cell differentiation, CD2 is inhibited in migration-positive B cells comparatively.

The higher tdTomato signal was also seen in NK cells of CD2-tdTomato-transgenic lines (**Figure 5**) and it indicated that Cre-activation worked under Tamoxifen induction.

The previous study reported that tdTomato expression was nearly absent in peripheral blood, and minimally expressed in splenic macrophages, granulocytes, monocytes and dendritic cells of hCD2-tdTomato-transgenic lines (27). Similar to other CD2-iCre discussed above, splenic myeloid cells may express CD2 at very low levels, almost undetectable levels, but this may nevertheless be sufficient to cause occasional all-or-none Cre recombination events (**Figure 6**). In contrast to other myeloid cell types, maximal CD2-Cre activity in blood monocytes might suggest that the potential impact of altered monocyte function on any CD2-Cre-related phenotypes should be carefully considered. Additionally, there is a variation between male and female mice in the proportion of CD2^+^tdTomato^+^ cells in different cells from different tissues. One potential explanation for this discrepancy is that CD2 expression levels may differ between male and female mice, potentially influenced by genetic and hormonal factors. A study identified a polymorphic estrogen receptor binding site that regulates CD2 expression, contributing to female-specific differences in T cell populations (42). This finding suggests that estrogen receptor binding can modulate CD2 expression, leading to sex-specific variations.

In summary, CD2-driven Cre is expressed in lymphocyte-derived and peripheral T/B cells. Furthermore, the high tdTomato fluorescence observed in blood monocytes indicates this line has unique utility for CD2-related research, including monocyte infiltration. Importantly, no leakage of Cre protein was detected from these tissues, as no tdTomato expression was observed in the absence of tamoxifen. In summary, we established *Cd2-CreERT2* knock-in mice that induced Cre recombination in all lymphoid and myeloid cells. This study provides valuable insights into the activity patterns of the CD2-Cre transgene across various cell populations. These mice represent a valuable resource for temporal genetic manipulation, particularly in T cells, with potential therapeutic applications.

## Data Availability

The original contributions presented in the study are included in the article/**Supplementary Material**. Further inquiries can be directed to the corresponding authors.

## Supplementary material

Supplementary Figure 1 Diagram of transgene knock-in event at the *Cd2* locus, genotyping scheme for F0/F1 generation *Cd2-CreERT2* mice and representative electrophoresis. **(A)** Schematic depicting the generation of endogenous *Cd2-CreERT2* knock-in reporter mouse line. **(B)** Genotyping scheme for F0/F1 generation *Cd2-CreERT2* knock-in mice. With P1/P2 primers, a 5.8 kb fragment is expected from the 5’arm homologous recombination positive genome, while none from the wild-type genome. With P3/P4 primers, a 2.9 kb fragment is expected from the 3’arm homologous recombination positive genome, while a 7.3 kb fragment from the wild-type genome. **(C)** Representative gel image show PCR amplicons from the 5’ or 3’ homology arm of founders using primers P1-P4. WT-wild-type mice samples, No.1-6-heterozygous knock-in mice samples, Marker left- 5’ arm PCR products, Marker right-3’ arm PCR products, M-1kb DNA marker (Thermo Code No.SM0311).

Supplementary Figure 2 Genotyping scheme for F2 generation *Cd2-CreERT2* knock-in mice, and representative electrophoresis. **(A)** Genotyping scheme for F2 generation *Cd2-CreERT2* knock-in mice. The primer pairs (P5/P6 or P5/P7) were used to genotype and check the precise integration site. The forward primer P5 was shared and was located upstream of exon 5, while the reverse primer P7 was located inside the *CreERT2* sequence to differ from WT (primer P6). **(B)** Representative gel image showing PCR results for F2 offspring amplified by primers P5-P7. Heterozygous 1^#^ -4^#^ showed the positive band with the expected size of 410 bp, as well as the WT-band (366 bp). M:100 bp Plus DNA Ladder (Transgene Code No. BM311-02)

Supplementary Figure 3 Southern blot analysis of the genomic DNA of WT and *Cd2-CreERT2* knock-in mice. **(A)** Localization of the restriction enzyme sites, digested fragments and the Probe-Cre on the *Cd2-CreERT2* knock-in allele. **(B)** Southern blot of genomic DNA digested with BglII or AflII.

Supplementary Figure 4 Evaluation of endogenous *Cd2* expression in WT and heterozygous *Cd2-CreERT2* knock-in mice. **(A)** Quantitative RT-PCR analysis of *Cd2* mRNA expression level in C57BL/6 mice and *Cd2-CreERT2* mice. **(B)** Western blot band images of CD2 and GAPDH protein in C57BL/6 mice and *Cd2-CreERT2* mice. **(C)** Quantification of CD2 protein expression level in C57BL/6 mice and *Cd2-CreERT2* mice.

Supplementary Figure 5 Immunofluorescence images of the mesenteric lymph node of the WT and homozygous *Cd2-CreERT2* mice.

Supplementary Figure 6 TdTomato fluorescence signals in the tissues of *Cd2-CreERT2*-inducible tdTomato mice. **(A)** Brain, **(B)** Lung, **(C)** Heart, **(D)** Thymus, **(E)** Spleen, **(F)** Kidney, **(G)** Pancreas, **(H)** Liver, **(I)** Stomach and intestine, **(J)** Testis, **(K)** Uterus, **(L)** Mesenteric lymph node.
