# Supplementary material for: Generation and characterization of a tamoxifen-inducible lineage tracing tool Cd2-P2A-CreERT2 knock-in mice
Source: Front Immunol. 2025 Mar 10;16:1482070. doi: 10.3389/fimmu.2025.1482070 (PMC11931051; doi:10.3389/fimmu.2025.1482070)
Supplement: Supplementary Figure 1 — Diagram of transgene knock-in event at the Cd2 locus, genotyping scheme for F0/F1 generation Cd2-CreERT2 mice and representative electrophoresis. (A) Schematic depicting the generation of endogenous Cd2-CreERT2 knock-in reporter mouse line. (B) Genotyping scheme for F0/F1 generation Cd2-CreERT2 knock-in mice. With P1/P2 primers, a 5.8 kb fragment is expected from the 5’arm homologous recombination positive genome, while none from the wild-type genome. With P3/P4 primers, a 2.9 kb fragment is expected from the 3’arm homologous recombination positive genome, while a 7.3 kb fragment from the wild-type genome. (C) Representative gel image show PCR amplicons from the 5’ or 3’ homology arm of founders using primers P1-P4. WT-wild-type mice samples, No.1-6-heterozygous knock-in mice samples, Marker left- 5’ arm PCR products, Marker right-3’ arm PCR products, M-1kb DNA marker (Thermo Code No.SM0311). [file Image1.pdf]

Supplementary material

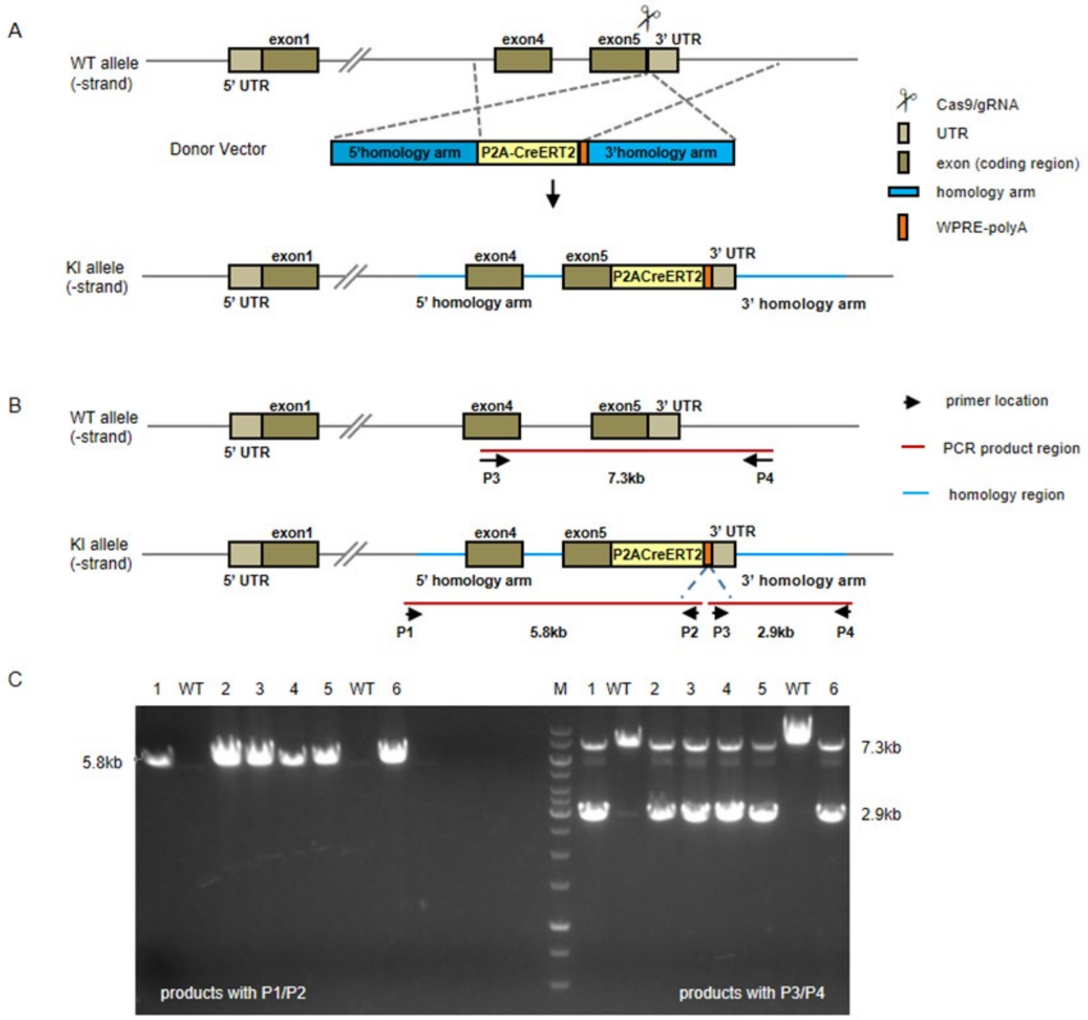

Supplementary Figure 1. Diagram of transgene knock-in event at the *Cd2* locus, genotyping scheme for F0/F1 generation *Cd2-CreERT2* mice and representative electrophoresis.

(A) Schematic depicting the generation of endogenous *Cd2-CreERT2* knock-in reporter mouse line. **(B)** Genotyping scheme for F0/F1 generation *Cd2-CreERT2* knock-in mice. With P1/P2 primers, a 5.8 kb fragment is expected from the 5'arm homologous recombination positive genome, while none from the wild-type genome. With P3/P4 primers, a 2.9 kb fragment is expected from the 3'arm homologous recombination positive genome, while a 7.3 kb fragment from the wild-type genome. **(C)** Representative gel image show PCR amplicons from the 5' or 3' homology arm of founders using primers P1-P4. WT-wild-type mice samples, No.1-6-heterozygous knock-in mice samples, Marker left- 5' arm PCR products, Marker right-3' arm PCR products, M-1kb DNA marker (Thermo Code No.SM0311).

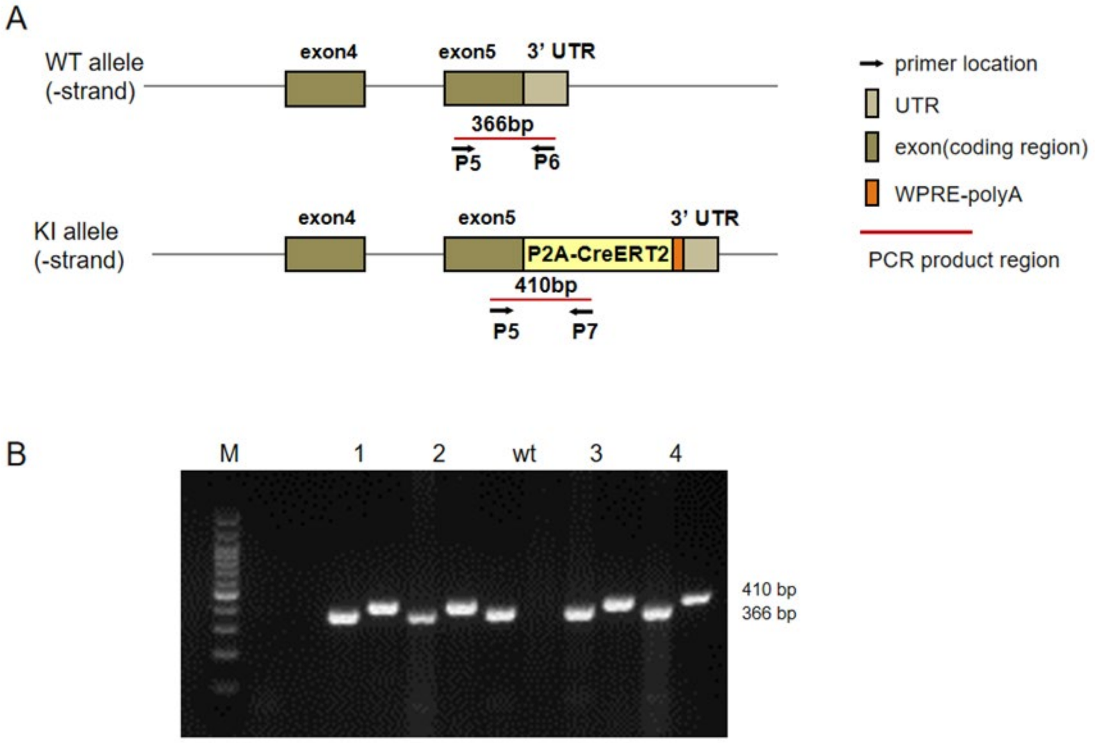

Supplementary Figure 2. Genotyping scheme for F2 generation *Cd2-CreERT2* knock-in mice, and representative electrophoresis.

(A) Genotyping scheme for F2 generation *Cd2-CreERT2* knock-in mice. The primer pairs (P5/P6 or P5/P7) were used to genotype and check the precise integration site. The forward primer P5 was shared and was located upstream of exon 5, while the reverse primer P7 was located inside the *CreERT2* sequence to differ from WT (primer P6). (**B**) Representative gel image showing PCR results for F2 offspring amplified by primers P5-P7. Heterozygous $1^{\#}$-$4^{\#}$ showed the positive band with the expected size of 410 bp, as well as the WT-band (366 bp). M:100 bp Plus DNA Ladder (Transgene Code No. BM311-02)

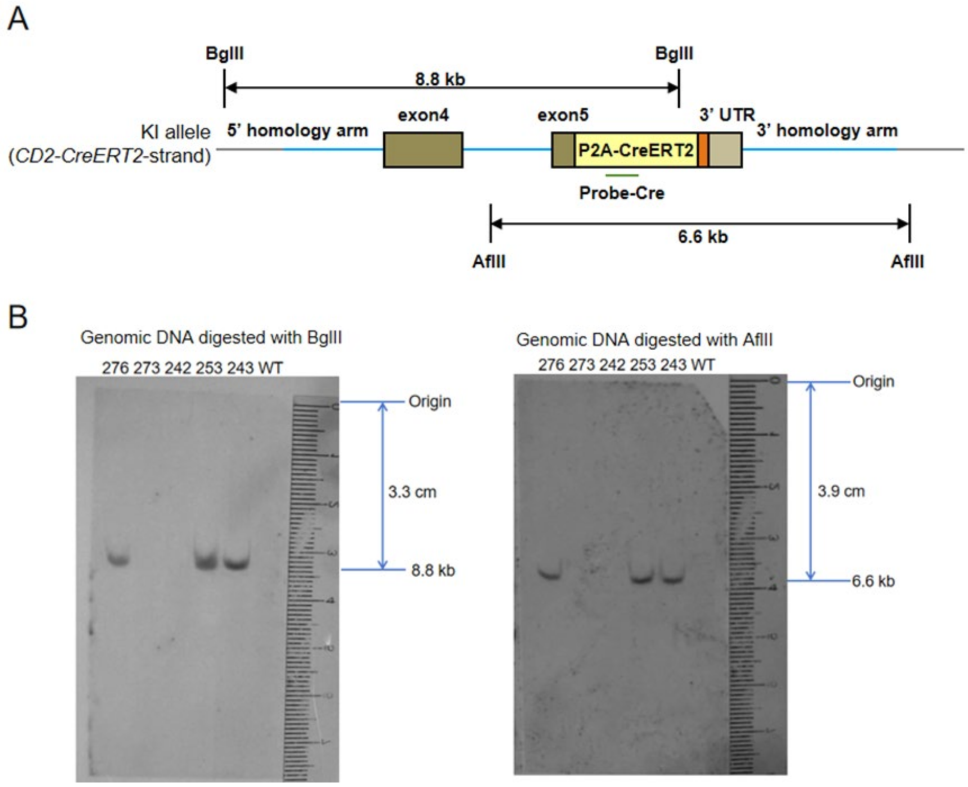

Supplementary Figure 3. Southern blot analysis of the genomic DNA of WT and *Cd2-CreERT2* knock-in mice.

(A) Localization of the restriction enzyme sites, digested fragments and the Probe-Cre on the *Cd2-CreERT2* knock-in allele. **(B)** Southern blot of genomic DNA digested with BglII or AflII.

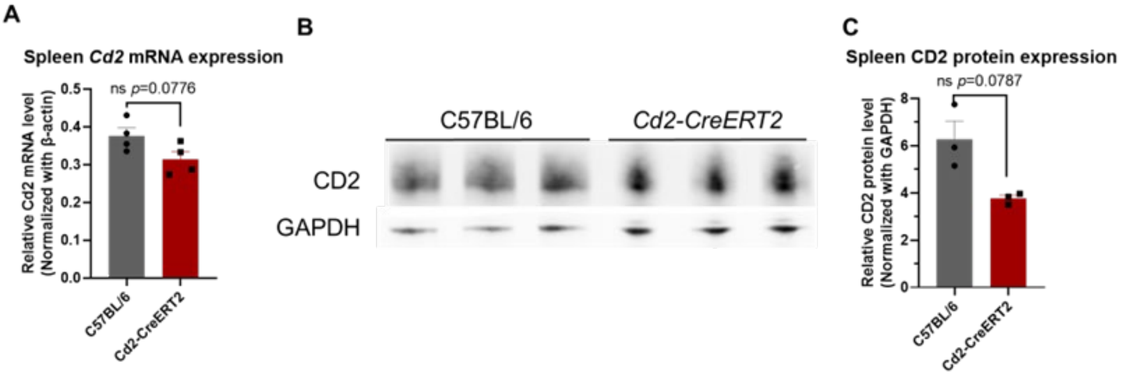

Supplementary Figure 4. Evaluation of endogenous *Cd2* expression in WT and heterozygous *Cd2-CreERT2* knock-in mice.

(A) Quantitative RT-PCR analysis of *Cd2* mRNA expression level in C57BL/6 mice and *Cd2-CreERT2* mice. **(B)** Western blot band images of CD2 and GAPDH protein in C57BL/6 mice and *Cd2-CreERT2* mice. **(C)** Quantification of CD2 protein expression level in C57BL/6 mice and *Cd2-CreERT2* mice.

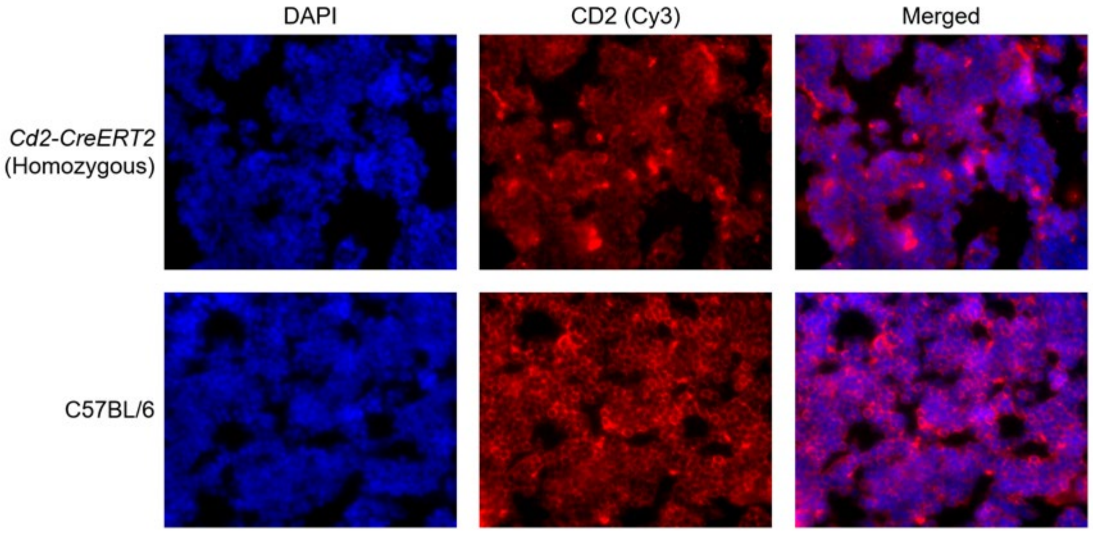

Supplementary Figure 5. Immunofluorescence images of the mesenteric lymph node of the WT and homozygous *Cd2-CreERT2* mice.

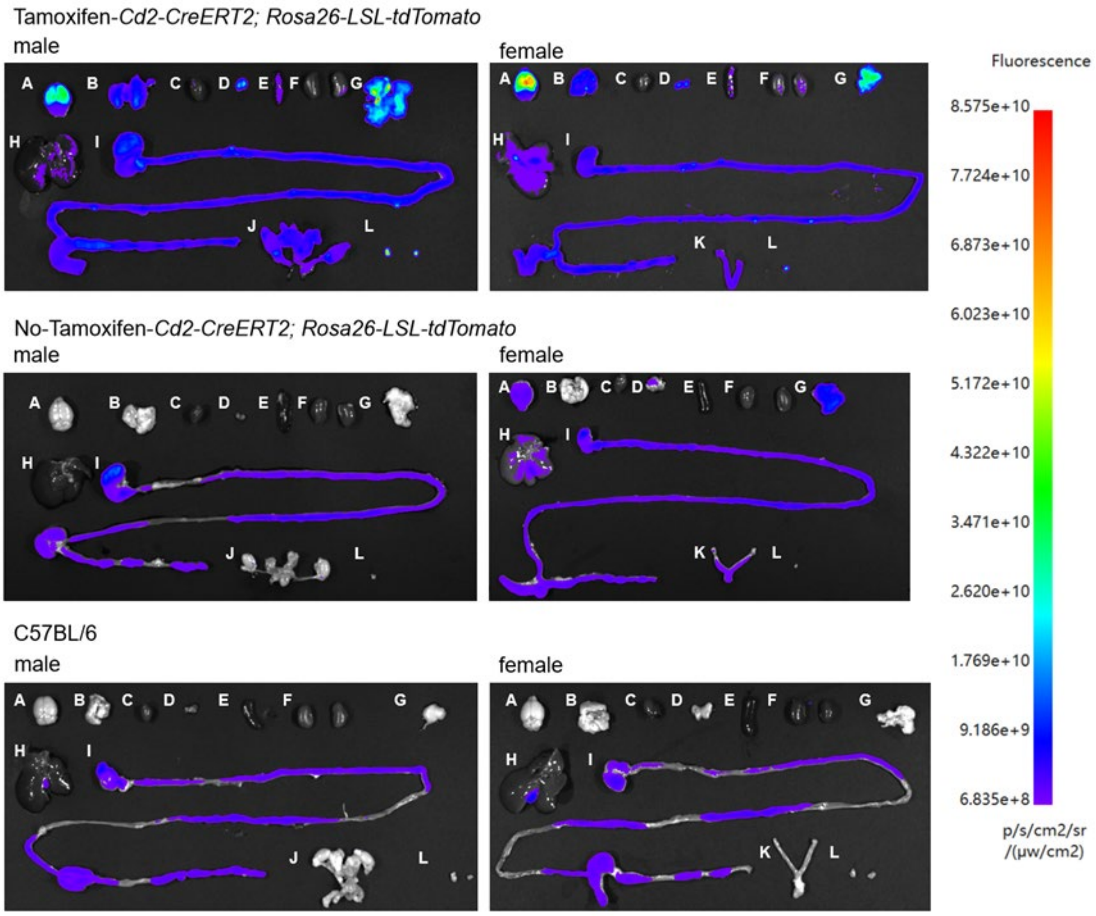

Supplementary Figure 6. TdTomato fluorescence signals in the tissues of *Cd2-CreERT2*-inducible tdTomato mice.

(A) Brain, **(B)** Lung, **(C)** Heart, **(D)** Thymus, **(E)** Spleen, **(F)** Kidney, **(G)** Pancreas, **(H)** Liver, **(I)** Stomach and intestine, **(J)** Testis, **(K)** Uterus, **(L)** Mesenteric lymph node.
